# Public Health Impact After the Introduction of PsA-TT: The First 4 Years

**DOI:** 10.1093/cid/civ499

**Published:** 2015-11-09

**Authors:** Fabien V. K. Diomandé, Mamoudou H. Djingarey, Doumagoum M. Daugla, Ryan T. Novak, Paul A. Kristiansen, Jean-Marc Collard, Kadidja Gamougam, Denis Kandolo, Nehemie Mbakuliyemo, Leonard Mayer, James Stuart, Thomas Clark, Carol Tevi-Benissan, William A. Perea, Marie-Pierre Preziosi, F. Marc LaForce, Dominique Caugant, Nancy Messonnier, Oladapo Walker, Brian Greenwood

**Affiliations:** 1 Centers for Disease Control and Prevention, Atlanta, Georgia; 2 Inter-country Support Team for West Africa, World Health Organization, Ouagadougou, Burkina Faso; 3 Centre de Support en Santé International, N'Djamena, Chad; 4 World Health Organization, Collaborating Center for Reference and Research on Meningococci, Norwegian Institute of Public Health, Oslo, Norway; 5 Centre de Recherche Médicale et Sanitaire, Niamey, Niger; 6 Centre de Support en Santé International, N'Djamena, Chad; 7 Faculty of Infectious and Tropical Diseases, London School of Hygiene and Tropical Medicine, United Kingdom; 8 World Health Organization, Regional Office for Africa, Brazzaville, Republic of Congo; 9 Department of Pandemic and Epidemic Diseases, World Health Organization, Geneva, Switzerland; 10 Meningitis Vaccine Project, PATH, Ferney-Voltaire, France; 11 Meningitis Vaccine Project, Department of Immunization, Vaccines and Biologicals, World Health Organization, Geneva, Switzerland; 12 Serum Institute of India, Ltd, Pune

**Keywords:** meningococcal group A, PsA-TT, vaccine evaluation framework, disease incidence, Africa meningitis belt

## Abstract

***Background.*** During the first introduction of a group A meningococcal vaccine (PsA-TT) in 2010–2011 and its rollout from 2011 to 2013, >150 million eligible people, representing 12 hyperendemic meningitis countries, have been vaccinated.

***Methods.*** The new vaccine effectiveness evaluation framework was established by the World Health Organization and partners. Meningitis case-based surveillance was strengthened in PsA-TT first-introducer countries, and several evaluation studies were conducted to estimate the vaccination coverage and to measure the impact of vaccine introduction on meningococcal carriage and disease incidence.

***Results.*** PsA-TT implementation achieved high vaccination coverage, and results from studies conducted showed significant decrease of disease incidence as well as significant reduction of oropharyngeal carriage of group A meningococci in vaccinated and unvaccinated individuals, demonstrating the vaccine's ability to generate herd protection and prevent group A epidemics.

***Conclusions.*** Lessons learned from this experience provide useful insights in how to guide and better prepare for future new vaccine introductions in resource-limited settings.

To reduce the frequency of deadly outbreaks of meningitis in Africa, the Meningitis Vaccine Project (MVP) developed a novel group A meningococcal conjugate vaccine (PsA-TT), manufactured by the Serum Institute of India, Ltd. Licensed in India in 2009, PsA-TT was prequalified by the World Health Organization (WHO) in June 2010 and initially introduced in September 2010, when it was given to 1- to 29-year-olds who resided in 3 highly endemic/epidemic countries in West Africa (Burkina Faso, Mali, and Niger) with the support of the Global Alliance for Vaccines and Immunization (Gavi). Between 2011 and 2013, PsA-TT was introduced in a phased approach to parts or all of 9 additional hyperendemic countries (Chad, Cameroon, Nigeria, Benin, Ghana, Senegal, Sudan, Gambia, and Ethiopia), representing a target population of 150 million people vaccinated [1].

To coordinate the international effort to evaluate PsA-TT effectiveness in Africa, the WHO Inter-country Support Team for West Africa (IST-WA) established a framework to evaluate results. Several evaluation studies were planned in coordination with international public health partners. This paper describes the efforts to evaluate PsA-TT effectiveness, analyzes the results of the evaluation studies obtained so far, discusses the main constraints and challenges faced, and reviews lessons learned.

## EVALUATION FRAMEWORK: GOALS, OBJECTIVES, AND METHODS

The postlicensure PsA-TT evaluation studies, undertaken in early-introducer countries (Burkina Faso, Mali, Niger, and Chad), aimed to demonstrate the effectiveness and public health impact of PsA-TT under field conditions following its first introduction in Africa. Six objectives were identified:

1. Monitoring vaccination coverage following the mass vaccination campaigns. Vaccination coverage estimates, which may be influenced by local implementation issues, are essential for the interpretation of measurements of vaccine impact.
2. Estimating the reduction in disease burden (incidence and prevalence) by analyzing national surveillance data collected before and after PsA-TT introduction.
3. Estimating vaccine effectiveness on carriage and herd protection through pre- and postvaccine studies.
4. Monitoring the immune response to the vaccine.
5. Undertaking cost and benefit studies associated with vaccination.
6. Evaluating the impact of the vaccine's introduction on a country's national immunization program.

Not all countries, or regions within a country, had the capacity to undertake vaccine evaluation studies.

Enhanced meningitis surveillance (EMS) was implemented in meningitis belt countries several years before the introduction of PsA-TT, using standardized operating procedures developed by the WHO Regional Office for Africa (AFRO) and WHO headquarters in Geneva [2]. Additional efforts to strengthen meningitis case-based surveillance capacity prior to the introduction of PsA-TT depended on specific evaluation needs and resources. These resources included a health infrastructure able to detect suspected meningitis cases and collect cerebrospinal fluid (CSF) samples; a well-functioning system for transporting specimens to district, regional, and/or national laboratories; laboratory capacity for Gram staining, rapid testing of CSF samples using culture and a latex seroagglutination test; district capacity to accurately monitor and evaluate the vaccination campaign; and an ability to implement a system to detect, investigate, manage, and document adverse events following immunization with PsA-TT.

In Burkina Faso, substantial efforts were made since 2009 to move the existing EMS system toward a stronger, laboratory-confirmed case-based surveillance system with financial and technical support from the Centers for Disease Control and Prevention (CDC) and WHO. The EMS collects demographic case data and CSF results obtained from a proportion of patients, as recommended by the WHO EMS standard operating procedures [2]. Before the 2010 national PsA-TT mass campaign, revised case-based surveillance standard operating procedures for epidemic-prone diseases were implemented nationwide, following an initial pilot phase that concentrated on 10 districts. The scale-up of meningitis case-based surveillance in Burkina Faso was accompanied by significant efforts to improve specimen collection and transport to the national reference laboratory, pathogen confirmation with real-time polymerase chain reaction techniques, introduction of a unique identifier number to link laboratory results to patients' information, and improvement of data management and data quality (completeness and timely reporting) [3, 4]. Similar efforts were undertaken in 3 health regions in Chad during March–June 2012, with support from the MenAfriCar consortium, where 1- to 29-year-olds had been vaccinated with PsA-TT in December 2011, except for one district [5]. In addition to these efforts to strengthen a move toward strong, laboratory-confirmed, case-based surveillance in a restricted number of countries, WHO and its partners continued to support the existing EMS throughout the meningitis belt through refresher training, provision of laboratory supplies, and monitoring and supervision.

Special studies were undertaken in Burkina Faso, Niger, and Chad to measure the impact of PsA-TT on disease incidence, meningococcal carriage, and herd protection [4–8]. Routinely collected meningitis surveillance data were analyzed in the 3 countries [4–6]. In Burkina Faso, cross-sectional meningococcal carriage studies were undertaken in a representative sample of the 1- to 29-year-old population before and after PsA-TT campaigns by the Ministry of Health with technical and financial support from public health international partners including the Norwegian Institute of Public Health, the CDC in Atlanta, and the WHO Inter-country Support Team for West Africa [7, 8]. A total of 45 847 oropharyngeal samples were analyzed [7, 8]. Similar cross-sectional surveys were conducted in an age-stratified sample of population in a rural area in Chad before and after PsA-TT introduction, where a total of 10 277 samples were analyzed [5].

Three methods were used to estimate PsA-TT vaccination coverage:

1. Administrative coverage. The fraction of the district/health catchment area population belonging to the target 1- to 29-year-old age group was calculated and adjusted for population growth. Administrative coverage was calculated by dividing the number of people vaccinated (numerator) by the vaccination target (denominator).
2. Independent monitoring. The independent monitoring approach aimed to detect nonvaccinated at-risk groups (specific age groups or geographic areas) to create innovative ways of reaching these populations. Independent monitoring was conducted during the campaign (in-process monitoring) and during the first week after the completion of the campaign (end-process monitoring). Monitoring was performed by independent monitors using standardized forms. Two settlements in a catchment area were selected randomly by the monitor, and 5 households were visited in each settlement. In each household visited, information was collected systematically on the following variables:
  - Number of people physically seen in a household by age group.
  - Total number of people with a vaccination card.
  - Total number of people not vaccinated.
  - Reason for nonvaccination (absence from the area, active decision not to be vaccinated, vaccination post too far away, long waiting times, no vaccinator at the vaccination post, and/or lack of information).
  - Information sources about the vaccine.
  - Awareness of potential side effects from the vaccine.
  In addition to the household visits, a group of twenty 1- to 29-year-olds was selected randomly and asked similar information about vaccination as listed above. Having a vaccination card that indicated that PsA-TT had been given was considered as proof of vaccination. Vaccination coverage was calculated by dividing the number of people with vaccination cards (numerator) by the total number of people physically seen (denominator).
3. Random coverage survey. In addition to the aforementioned approaches, random coverage surveys were conducted at least 1 month after the vaccination campaigns. Two methods were used to conduct these surveys: the stratified-cluster survey method, recommended by the WHO Expanded Programme on Immunization (EPI) [9, 10]; and the Lot Quality Assurance Sampling (LQAS) method [11, 12]. The former provides age-group-specific, sex, and subnational vaccination coverage estimates. Countries adapted this method to their specific context. The LQAS method was used to assess if eligible population subgroups, defined as Lots (age groups, sex, districts, etc) had achieved minimum acceptable vaccination coverage of 70%. Sampling frames were derived from a previous population census and sample sizes of subjects were calculated and divided into clusters with the desired precision of 5%, assuming a coverage of 85%, a 95% confidence interval, and a design effect of 2. Lower and upper coverage thresholds were set and decision values were defined in accordance with LQAS methods. Probability proportionate to population size was used to select clusters in each district, as described in 2-stage cluster sampling methods [10].

## VACCINE IMPACT ON EPIDEMIC MENINGITIS

It was anticipated that, as with other conjugate vaccines, PsA-TT would have 2 direct effects: (1) protection against invasive meningococcal disease and (2) reduction of nasopharyngeal carriage of meningococci. In addition, by preventing carriage, the vaccine should reduce transmission of the meningococcus, providing indirect protection to nonvaccinated subjects (herd protection) and halting or preventing an epidemic.

## DISEASE BURDEN REDUCTION

**Figure 1.**
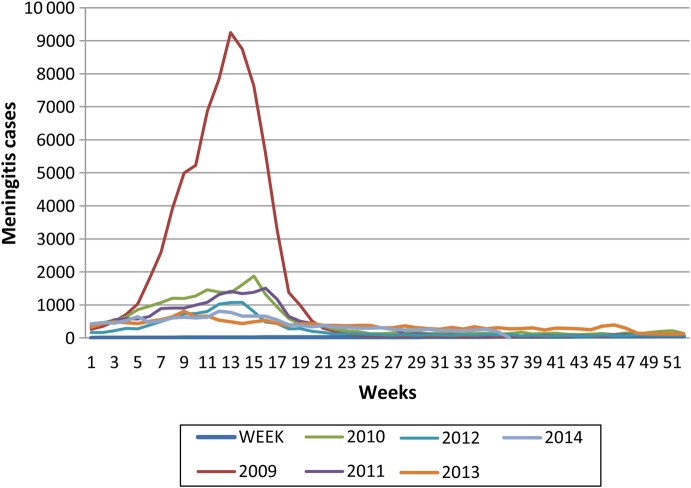
Incidence of meningitis in the African meningitis belt, 2009–2014. Source: www.meningvax.org.

Because PsA-TT was licensed only on safety and immunogenicity data, as with some other conjugate vaccines, measurement of its postmarketing effectiveness was considered a public health priority. Review of meningitis weekly incidence data, obtained from the WHO Meningitis Weekly Bulletin, shows a marked decline in the overall incidence of meningitis and a reduction in the size of outbreaks in the African meningitis belt since the time of the first introduction of PsA-TT (Figure 1). In Burkina Faso, where PsA-TT was first introduced in 2010 through a countrywide, mass vaccination campaign that reached 11.4 million people aged 1–29 years, national population-based surveillance data were collected for 14 years (1997–2010) before and for 1 year after PsA-TT introduction (2011) [4]. Population estimates from the Burkina Faso National Institute of Statistics and Demography were used to calculate the following parameters during the first year after PsA-TT introduction and to compare epidemic and nonepidemic years before vaccine implementation: incidence of suspected and probable cases of meningococcal meningitis by age group, mortality rate, group-specific meningococcal disease incidence rates, and risk of death [4]. Statistically significant decreases in all causes of meningitis, meningitis epidemics, and group A meningococcal disease were found [4]. Decreases in suspected and probable meningitis cases during the first year after PsA-TT introduction were noted compared with incidence rates during the lowest epidemic years before vaccine introduction [4]. In Niger, where PsA-TT was introduced in 2 phases, the last quarters of 2010 and 2011, similar analysis of routinely collected meningitis surveillance and laboratory data of the period 2008–2011 was conducted and showed a decline of incidence of group A cases as observed in Burkina Faso, despite the fact that only 11 of the 42 districts of the country had been vaccinated after the first phase of the PsA-TT campaign (around 50% of the target population for vaccination) [6]. The observed disappearance of group A cases from the whole of Niger is likely related to both a natural dynamic of meningitis epidemics in the belt and massive vaccination with polysaccharide A/C vaccines in previous years [6, 13]. In Burkina Faso, it was not possible to compare vaccinated and unvaccinated areas, as the whole country was covered within a short period of time, and “before and after” analyses of vaccine impact can be affected by the changes in disease incidence over time that are independent of vaccine introduction [6, 13]. PsA-TT was introduced in Chad in December 2011, but the initial mass vaccination campaign covered only 3 regions (N'Djamena, Chari-Baguirma, and Mayo-Kebbi) [5]. Vaccination of the rest of the country continued the following year. This phased introduction provided an opportunity to measure disease incidence in vaccinated and unvaccinated population at the same time [5]. Comparison of the incidence of meningitis in the 3 regions where PsA-TT campaigns had taken place with the areas that had not been vaccinated showed that the epidemic had been halted in the vaccinated areas, as well as a statistically significant difference between areas in the incidence of all cases of meningitis of 94% [5]. Analysis of routinely collected surveillance data in Burkina Faso, Niger, and Chad show strong evidence for a high level of short-term vaccine effectiveness [4–6].

## IMPACT ON DISEASE TRANSMISSION AND HERD PROTECTION

The ability of PsA-TT to reduce disease transmission and to generate herd protection was assessed by carriage studies conducted in Burkina Faso and Chad. The overall results are summarized in Table 1.

**Table 1.** Group A Meningococcal Carriage Before and After PsA-TT Campaigns in Burkina Faso and Chad

Country	Prevaccination			Postvaccination			Difference in Prevalence, % (aOR [95% CI])	*P* Value
	Sample, No.	Carriage, No.	Prevalence, %	Sample, No.	Carriage, No.	Prevalence, %		
Burkina Faso	20 326	80	0.39	22 093	0	0	NA	.003
Chad	998^a^	6	0.6	5001^b^	1	0.02	98 (0.019 [.002–.138])	
	4278^c^	32	0.7					

Source: [5, 7, 8].

Abbreviations: aOR, adjusted odds ratio; CI, confidence interval; NA, not applicable.

^a^ First survey conducted September–November 2010 (13–15 months before vaccination).

^b^ Third survey, conducted April–June 2012 (4–6 months after vaccination).

^c^ Second survey, conducted August–October 2011 (2–4 months before vaccination).

In Burkina Faso, the prevalence of meningococcal carriage was measured in 3 geographically distinct districts before the introduction of PsA-TT [7]. The overall baseline prevalence of meningococcal carriage was 3.98%, with the highest carriage rates being found among 15- to 19-year-old males and 10- to 14-year-old females, in rural districts, and during the dry season [7]. Group Y was dominant (2.28%), followed by group X (0.44%), group A (0.39%), and group W 0.35% [7]. Patterns of carriage varied between surveys, but no group A carrier was found after PsA-TT introduction in Burkina Faso, even among those who were not vaccinated because they were too young or old at the time of vaccination or had otherwise missed the vaccination campaign [8]. In Chad, cross-sectional meningococcal carriage surveys were conducted in the area where the first PsA-TT campaigns took place before and after vaccine introduction [5]. The prevalence of group A carriage dropped from 0.75% to 0.02% after PsA-TT introduction [5]. In Chad, carriage of group A meningococci fell after PsA-TT introduction in both vaccinated and unvaccinated subjects. This is consistent with the meningitis herd protection observed in both Burkina Faso and Chad [4, 5].

## CHALLENGES IN MEASURING PSA-TT EFFECTIVENESS

Since the first introductions in 2010, measurements of the effectiveness of PsA-TT have been conducted through the analysis of routinely collected surveillance data before and after vaccine introduction and through implementation of cross-sectional meningococcal carriage surveys. Extensive investments in surveillance and laboratory capacities were needed in countries such as Burkina Faso and Chad. The success of PsA-TT introduction, combined with the efforts to strengthen meningitis surveillance with strong laboratory confirmation of cases, has revealed changes in the epidemiologic pattern of meningococcal disease: cases of group A are disappearing, and consequently there has been an increase in the proportion of cases due to other groups and other bacteria (group W meningococci and pneumococci, respectively) [4–6, 14]. Therefore, the key new challenges in measuring PsA-TT long-term effectiveness include the need to maintain a sustainable laboratory-confirmed meningitis surveillance system to accurately detect cases to use as a platform for vaccine evaluation (including special studies) and finding ways to maintain high vaccination coverage, either through follow-up campaigns or introduction of PsA-TT into the routine EPI. Despite successful deployment of PsA-TT and its demonstrated short-term impact on epidemic meningitis, questions remain about its longer-term impact. These include the duration of protection; whether there will be an emergence or spread of other groups, serotypes, or other pathogens; how to determine correlates of protection and kinetics of vaccine-induced immunity; and what the immune response is in vaccine failure. We also need to better understand the mechanisms that underlie indirect protection offered by PsA-TT as well as use modeling to inform both short- and long-term vaccination strategies [15]. These methodological and programmatic challenges must be overcome to assess the long-term effectiveness of PsA-TT. This long-term assessment will need to include the use of PsA-TT in infants as part of EPI, following prequalification of the PsA-TT pediatric indication by WHO.
